# BRAF *V600E* mutation in papillary thyroid carcinoma: it’s relation to clinical features and oncologic outcomes in a single cancer centre experience

**DOI:** 10.1530/EC-21-0410

**Published:** 2021-11-03

**Authors:** Mahmoud Al-Masri, Tawfiq Al-Shobaki, Hani Al-Najjar, Rafal Iskanderian, Enas Younis, Niveen Abdallah, Abdelghani Tbakhi, Hussam Haddad, Mohammad Al-Masri, Zeinab Obeid, Awad Jarrar

**Affiliations:** 1 Department of Surgery, King Hussein Cancer Center, Amman, Jordan; 2 Department of Internal Medicine, Endocrine, King Hussein Cancer Center, Amman, Jordan; 3 Department of Pathology & Laboratory Medicine, King Hussein Cancer Center, Amman, Jordan; 4 Department of Cell Therapy & Applied Genomics, King Hussein Cancer Center, Amman, Jordan; 5 School of Medicine, University of Jordan, Amman, Jordan

**Keywords:** thyroid cancer, papillary, neoplasms, mutation, Middle East

## Abstract

**Purpose:**

This study focuses on the oncologic influence of BRAF *V600E* mutations in a cohort of Middle Eastern papillary thyroid carcinoma (PTC) patients treated at a single centre. We tested the association of BRAF V600E mutation with papillary thyroid carcinoma at King Hussein Cancer Center.

**Methods:**

Patients with histologically confirmed PTC who underwent surgical treatment between 2006 and 2015 were included in this study. Oncological outcomes, both short- and long-termed, were collected.

**Results:**

A total of 128 patients (68% females) were included in this study with a mean age of 38 years (±13.8). The median follow-up period was 50 months. The BRAF *V600E* mutation was found in 71% of patients. The tumour size for patients with a negative BRAF V600E mutation was significantly larger in comparison to patients who tested positive for the mutation (3.47 cm vs 2.31 cm, respectively, *P* = 0.009). The two groups showed similar disease-free survival (DFS) rates; positive = 75% (median 43 months (0–168)) compared to 78% for the negative BRAF *V600E* mutation (median 38 months (3–142)) (*P* = 0.162, HR = 0.731) Furthermore, both groups showed similar overall survival rates, positive = 94.5% (median 56 months (0–228)) compared to 94.6% for the negative BRAF *V600E* mutation (median 43 months (3–157)) (*P* = 0.941, HR = 0.940).

**Conclusion:**

BRAF *V600E* mutation had no effect on loco-regional recurrence, distant metastasis, overall survival, or DFS. These findings may be attributed to geographic variations or reflect that BRAF *V600E* may only serve as an indicator of poor prognosis in high-risk group as such.

## Introduction

Papillary thyroid carcinoma (PTC) is the most prevalent subtype of thyroid cancer accounting for approximately 85–88% of cases. The incidence of PTC is rapidly increasing, in contrast to other subtypes (1). The increase in the incidence of PTC is attributed to several controversial perspectives like coincidental detection on imaging studies, abundance of clinical surveillance, improvements in diagnostic technologies, such as high-resolution thyroid ultrasound and fine-needle aspiration biopsy (4). Another contributor may be the increase in avid pathological sampling of seemingly benign specimens that were historically underdiagnosed (3).

Despite the high prevalence of well-differentiated thyroid carcinoma, mortality rates remained relatively stable (2, 4). Recurrence is common with a rate of 15% over the course of 10 years and approximately 10% mortality as a result of disease progression (5, 6). Prognosis and disease progression are fundamentally determined by the stage of the disease. However, the role of associated gene mutations on these parameters is unclear.

The majority of PTC are clinically inert with simple genetic makeup harbouring a few copy-number modifications. In terms of whole-exome sequencing, PTC holds one of the lowest mutation rates among cancers (7). The literature has pinpointed various PTC subtypes with mutually exclusive gene mutations that signal through the mitogen-activated protein kinase (MAPK) pathway (8, 9). BRAF, specifically BRAF *V600E,* is the most common mutation, accounting for 60% of these mutations in thyroid cancer, with the highest incidence in PTC. BRAF is a constituent of the MAPK (ERK) signalling pathway, which activates transcription factors that are vital for directing cell cycle and survival (10). Its clinical correlation with PTC has been extensively studied with contradictory results (11, 12, 13, 14). However, these mutations have been commonly linked to extra-thyroidal extension and lymph node metastasis.

We aim to assess the incidence and clinical influence of BRAF* V600E* mutation on a cohort of PTC patients treated at a single institute. The primary end points of this study are disease-free survival (DFS) and overall survival (OS).

## Methods

This is a retrospective chart review study approved by the Institutional Review Board (IRB) at King Hussein Cancer Center (KHCC). IRB (Ref: 15KHCC101). The KHCC IRB is guided by the principles described in the World Medical Association’s Declaration of Helsinki (1964) and its amendments. Due to the retrospective nature of the study and the lack of personal or clinical details of participants that compromise anonymity, individual informed consent was waived and the study was approved by King Hussein Cancer Center Institutional Review Board (IRB). The data sets used and/or analysed during the current study are available upon request to the corresponding author.

All patients with PTC who underwent hemithyroidectomy or total thyroidectomy with or without lymph node dissection between January 2006 and December 2015 were included in the study.

Based on our records, there are only 130 cases of PTC in our files. Two cases were excluded for other primary diagnoses namely gastric adenocarcinoma and second Hodgkin’s disease.

### Study cohort and tumour samples

Patients presenting with primary thyroid carcinomas, between 2006 and 2015, were retrospectively analysed.

In all cases, curative hemithyroidectomy or total thyroidectomy with or without neck dissection was performed. Radioiodine therapy was administered when indicated in accordance with institutional guidelines.

All patients were regularly followed up by physical examination, thyroid function tests (TFT) and neck ultrasonography every 6–12 months after primary surgery. Suspicious thyroid nodules or lymph nodes warranted ultrasound-guided fine-needle aspiration cytology (US-FNAC).

Tumour-node-metastasis (TNM) staging was defined based on the eighth edition of the American Joint Committee on Cancer (AJCC) staging system.

### Molecular testing for somatic genetic changes

All the retrieved hematoxylin and eosin (H&E) stained sections for the cohort cases were reviewed separately by two experienced histopathologists at the Endocrine Pathology Department within our institution. Both pathologists reviewed and confirmed the diagnoses of papillary thyroid carcinoma.

The most appropriate slide for BRAF molecular testing was determined based on the percentage of primary thyroid tumour and lymph node metastases if present. A cut-off point of 10% was deemed the minimal accepted tumour percentage on the selected slides. Five sections of approximately 5–10 µm thickness were sectioned from the formalin-fixed paraffin-embedded (FFPE) tumour tissue corresponding to the selected slides. Sectioned tissues were collected in Eppendorf tubes and labelled appropriately. The DNA was extracted and purified using the QIAamp® DNA Mini Kit (Qiagen). Samples were assessed for DNA concentration and purity using the NanoDrop® ND-1000 spectrophotometer. BRAF mutation testing was performed using therascreen® BRAF RGQ PCR Kit on the QIAGEN Rotor-Gene Q MDx instrument, designed to detect five somatic mutations in the BRAF gene including *V600E*, *V600E* complex (*V600Ec*), *V600D*, *V600K*, and *V600R*.

### Statistical analysis

Patients’ demographics, pathological data, and clinical outcomes were collected in a retrospective method. Data was analysed using software package SPSS 24. Results were expressed as medians and interquartile ranges (IQR) or mean and s.d.. Comparison between the two groups was performed using the χ^2^ test for categorical variables and the *t*-test for continuous variables. Survival functions were compared using the non-parametric Kaplan–Meier estimator. Clinical and pathological predictors of DFS and OS were analysed and multivariate Cox proportional-hazards models using software package SPSS 24. We analysed the data by Cox regression model for factors like gender and age for DFS and gender, family history, and age for OS. Significance was defined as *P*-value < 0.05. Statistically significant factors in univariate analysis were included in the multivariate model.

## Results

### Patient demographics

One hundred and twenty-eight patients were included in this study. The mean age was 38 years (±13.8) at the time of diagnosis. Forty-one patients (32%) were men and 87 patients (68%) were women. The cohort was followed for a median of 50 months post-surgical resection. No patients were lost for follow-up.

The mean size of the primary tumour was 2.6 cm (±2.2), 86% were stage I PTC tumours. BRAF *V600E* mutation was found in 91 patients (71%) out of 128 patients with classical PTC. Table 1 shows the characteristics of the 128 patients with conventional PTC included in the study.

**Table 1:** Clinicopathological characteristics.

	Variables	Total, *n* = 128 (%)
BRAF gene mutation	Positive Negative	91 (71.1) 37 (28.9)
Age	<55 years ≥55 years	113 (88.3) 15 (11.7)
Gender	Male Female	41 (32) 87 (68)
Extra-thyroidal extension	True False	32 (25) 96 (75)
Family history	Positive	9 (7)
Lymph node	Positive	68 (53)
Multi-Nodular goitre	Concomitant	12 (9.4)
Hashimoto thyroiditis	Concomitant	23 (18)
Pathology stage	I II III IV	110 (85.9) 12 (9.4) 1 (0.8) 5 (3.9)
Extent of surgery (total thyroidectomy)	Without neck dissection	50 (39.1)
	With neck dissection	77 (60.2)
Subtype of papillary thyroid cancer	Insular	1 (0.8)
	Follicular	4 (3.1)
	Capsular	11 (8.6)
	Classic	112 (87.5)
Tumour size (cm)	Mean (s.d.)	2.62 (2.17)
	Median (range)	2 (0–16)
Bilateral	True False	26 (20.3) 102 (79.7)
Multifocal	True False	54 (42.2) 74 (57.8)

### Association of BRAF V600E with clinicopathological features in PTCs

The various clinicopathological characteristics were compared between patients with PTC harbouring BRAF *V600E* mutation and those without (Table 2). The age and sex were not significantly different between the two groups (with or without BRAF *V600E* mutation). Patients with BRAF *V600E* positive were similar to BRAF *V600E* negative patients in regards to lymph node metastases (57.1% vs 43.2%, *P* = 0.36), extra-thyroidal extension (34.1% vs 35.0%, *P* = 0.90), positive family history (8.8% vs 2.7%, respectively, *P* = 0.22), multifocality (44.0% vs 37.8%, *P* = 0.52), and the extent of neck dissection. BRAF *V600E* negative tumours were significantly larger than the BRAF *V600E* positive (3.47 cm vs 2.31 cm respectively, *P* = 0.009).

**Table 2:** Patients characteristics based on BRAF status. Percentages are calculated out of total per column.

Variable vs BRAF Status	Total, *n* (%)	Positive, *n* (%)	Negative, *n* (%)	*P*-value
	128 (100)	91 (71.1)	37 (28.9)	
Gender				
Male	41 (32)	28 (30.8)	13 (35.1)	0.63
Female	87 (68)	63 (69.2)	24 (64.9)	
Positive family history	9 (7)	8 (8.8)	1 (2.7)	0.22
Age				
Mean (s.d.)	37.85 (13.75)	38.54 (13.58)	36.16 (14.22)	0.37
Primary tumour size (cm)				
Mean (s.d.)	2.62 (2.17)	2.31 (1.67)	3.47 (3.04)	0.009
Lymph node				
Positive	68 (53)	52 (57.1)	16 (43.2)	0.36
Negative	57 (44.5)	37 (40.7)	2 (5.4)	
Extra-thyroidal extension	44 (34.4)	31 (34.1)	13 (35)	0.90
Total thyroidectomy				
Without neck dissection	50 (39.1)	35 (38.5)	15 (40.5)	0.27
With neck dissection	77 (60.2)	56 (51.5)	21 (56.8)	
Concomitant Hashimoto	23 (18)	13 (14.3)	10 (27)	0.08
Hyperthyroidism	11 (8.6)	7 (7.7)	4 (10.8)	0.56
Multifocality	54 (42.2)	40 (44)	14 (37.8)	0.52
Subtype of papillary thyroid cancer				
Insular	1 (0.8)	0 (0.0)	1 (2.7)	0.276
Follicular	4 (3.1)	2 (2.2)	2 (5.4)	
Capsular	11 (8.6)	7 (7.7)	4 (10.8)	
Classic	112 (87.5)	82 (90.1)	30 (81.1)	
Multicentricity	26 (20.3)	19 (20.9)	7 (18.9)	0.80

**Figure 1:**
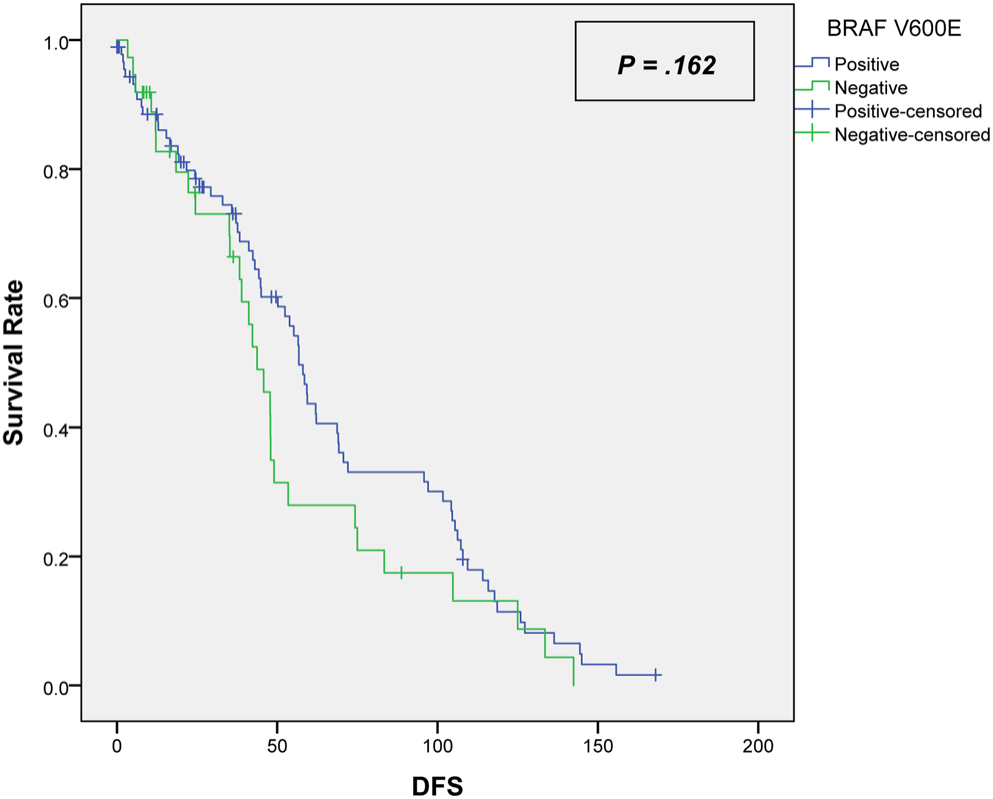
Kaplan–Meier curve for the disease-free survival.

**Figure 2:**
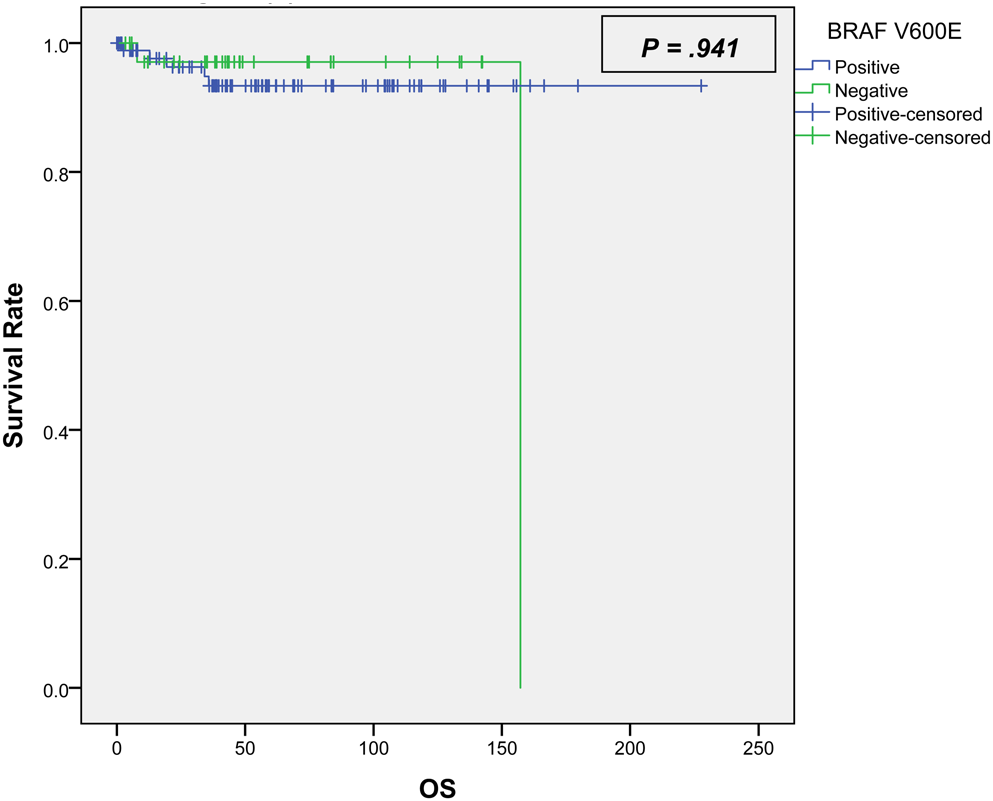
Kaplan–Meier curve for the overall survival.

With a median follow-up of 50 months, the two groups showed similar DFS (*P* = 0.162) (Fig. 1) and OS (*P* = 0.94) (Fig. 2).

### Risk factors affecting the disease-free and overall survival

To assess the effect of several risk factors on survival time in patients with PTC, DFS, and OS analysis were extended using univariate and multivariable Cox regression models. The BRAF *V600E* status, gender, family history, concomitant multinodular goiter, Hashimoto thyroiditis, multi-focality, multi-centricity, post-operative thyroglobulin level, and extent of neck dissection were tested. Female gender approached significance in association with improved DFS on univariate analysis (hazard ratio: 0.637, *P* = 0.052). No factors were associated with improved DFS on multivariable analysis (Table 3).

**Table 3:** Univariate analysis and multivariate Cox proportional hazards regression analysis for factors associated with disease-free survival.

Factor	Univariate analysis				Multivariate analysis			
	Hazard ratio	95% CI		*P*-value	Hazard ratio	95% CI		*P*-value
		Lower	Upper			Lower	Upper	
BRAF status	0.731	0.471	1.134	0.162				
Gender. female	0.637	0.404	1.004	0.052	0.687	0.435	1.087	0.109
Family history, negative	0.767	0.383	1.535	0.454				
Age	1.124	0.679	1.861	0.650				
Extra-thyroidal extension, negative	0.893	0.582	1.370	0.604				
Extent of neck dissection	1.975	1.262	3.090	0.003	1.910	1.216	3.000	0.005
Concomitant multinodular goitre	1.833	0.945	3.553	0.073				
Concomitant Hashimoto	0.842	0.513	1.380	0.495				
Multifocality	0.814	0.543	1.221	0.320				
Multicentricity	0.741	0.447	1.229	0.246				
Thyroglobulin level (post-op)	1.001	0.999	1.004	0.303				

In regards to OS (Table 4), patients < 50 years of age had significantly improved OS in unadjusted (hazard ratio 0.031, *P* = 0.001) and adjusted analysis (hazard ratio 0.037, *P* = 0.003).

**Table 4:** Univariate analysis and multivariate Cox proportional hazards regression analysis for factors associated with overall survival.

Factor	Univariate analysis				Multivariate analysis			
	Hazard ratio	95% CI		*P*-value	Hazard ratio	95% CI		*P*-value
		Lower	Upper			Lower	Upper	
BRAF status	0.940	0.182	4.866	0.941				
Gender, female	6.441	1.240	33.449	0.027	7.841	1.195	51.466	0.032
Family history, negative	5.660	1.036	30.934	0.045	2.367	.409	13.705	0.336
Age < 50 years	0.031	0.004	0.261	0.001	0.037	0.004	0.330	0.003
Extra-thyroidal extension	0.211	0.041	1.089	0.063				
Extent of neck dissection	1.100	0.230	5.268	0.905				
Concomitant multinodular goitre	0.617	0.072	5.286	0.659				
Concomitant Hashimoto	0.990	0.116	8.481	0.993				
Multifocality	0.736	0.149	3.649	0.708				
Multicentricity	0.474	0.087	2.591	0.389				
Thyroglobulin level (post-op)	1.002	0.996	1.007	0.556				

## Discussion

This study examined the clinicopathological factors associated with BRAF *V600E* mutation of patients treated at a single centre in Jordan. We explored the relationship between BRAF *V600E* and the oncological outcomes in PTC. The BRAF *V600E*, a point mutation at codon 600 of the BRAF gene, is a specific diagnostic and prognostic marker for PTC. BRAF results in constitutive activation of the BRAF kinase and uncontrolled proliferation of the MAP kinase signalling pathway.

Diagnostic age, male gender, multifocal tumour, and advanced TNM stage have been linked to worse prognosis in PTC (11, 12, 15, 16, 17, 18, 19). The literature reports variable findings in terms of prognostic features of BRAF mutations. Several studies have highlighted the presence of BRAF in clinically and histopathologically aggressive tumours (11, 19, 20). Xing *et al.* reported a significant association between BRAF mutations, adjacent structures invasion, and lymph node metastases (11). Moreover, the BRAF mutation was found to independently predict central lymph nodes metastases (19). A multivariate analysis of BRAF *V600E* mutation showed an independent correlation with worse outcomes and yielded survival curves demonstrating worse survival (21). BRAF may not reflect mortality and morbidity in early-stage PTC but rather serve as an indicator for more advanced cases. Therefore, since the majority of our patients displayed stage 1 PTC, survival and the majority of clinicopathological characteristics were comparable between BRAF *V600E* mutation-positive and -negative groups.

However, a number of large retrospective studies failed to validate an association between BRAF positive tumours and poor prognosis (13, 14, 22, 23, 24). Ito *et al*. investigated BRAF *V600E* mutation in 631 patients with papillary thyroid carcinoma with a median follow-up period of 83 months. The prevalence of BRAF *V600E* mutation was 38.4% and was not significantly linked to cases with high-risk biological features such as clinically apparent lymph node metastasis, massive extrathyroid extension, advanced age, distant metastases at surgery, and advanced stage. The DFS of patients with BRAF *V600E* positive mutation did not differ from those without mutation (22). These findings, in adjunct with our results, may justify that although BRAF *V600E* mutation may play a role in local development of the tumour, it does not always predict aggressive characteristics and poor prognosis.

Geographic variations may in fact be responsible for the variability in the literature with the difference in behaviour attributed to the racial background (24, 25). Studies in Asian populations noted no relationship between BRAF *V600E* mutation and regional lymph node metastases. Junliang *et al*. reported the outcome of 150 PTC cases of which 80% of primary tumours contained were positive for BRAF *V600E* mutation. Lymph node metastases was equally distributed in patients with or without BRAF *V600E* mutation (25).

The overall significance of BRAF mutation in PTC is unclear. In addition, the incidence and clinical impact of BRAF* V600E* mutation among PTC patients from Middle Eastern races remain unknown. To our knowledge, this is the first cohort study that investigated the relation between *BRAF V600E* mutation and PTC in the Middle Eastern population.

### Strengths and limitations

The strengths of this study are demonstrated through the length of follow-up, duplicate histopathology review, and availability of clinicopathological data. On the other hand, the main limitations of this study lie in its retrospective nature, small sample size, and majority of the cases being stage I. The small sample size may be a result of the pattern of case referral to a tertiary cancer center. Since PTC is common and treated surgically, the majority of cases are treated within the primary institution. As data are from a single center, these results may not be representative of all PTC cases in Jordan, and another limitation is that not all patients were treated surgically the same way.

## Conclusion

Despite the high percentage of PTCs harbouring BRAF *V600E* mutation, it did not affect lymph node involvement, locoreginal recurrence, distant metastases, OS, and DFS. Although BRAF has been commonly linked to poor prognosis and clinical aggressiveness, we believe that this may only be attributed to certain races and applicable in high-risk patients.

## Declaration of interest

The authors declare that there is no conflict of interest that could be perceived as prejudicing the impartiality of the research reported.

## Funding

This study was funded by King Hussein Cancer Center http://dx.doi.org/10.13039/100016168 (Grant Number: 15KHCC101).

## Ethics approval and consent to participate

This is a retrospective chart review study approved by the Institutional Review Board (IRB) at King Hussein Cancer Center (KHCC). IRB (Ref: 15KHCC101). The KHCC IRB is guided by the principles described in the World Medical Association’s Declaration of Helsinki (1964) and its amendments. Due to the retrospective nature of the study and the lack of personal identifiers that compromise anonymity, individual informed consent was waived. The study was approved by King Hussein Cancer Center Institutional Review Board (IRB). The data sets used and/or analysed during the current study are available from the corresponding author on reasonable request.

## Consent for publication

Informed consent was waived and the study was approved by King Hussein Cancer Center Institutional Review Board (IRB).

## Availability of data and materials

The data sets used and/or analysed during the current study are available from the corresponding author on reasonable request.

## Author contribution statement

All authors contributed to the study conception and design. Material preparation, data collection and analysis were performed by M M, H K N, T S, M M and R I. The first draft of the manuscript was written by T S, E Y, A T, H H, A J. All authors commented on previous versions of the manuscript. All authors read and approved the final manuscript.
